# Brain solute transport is more rapid in periarterial than perivenous spaces

**DOI:** 10.1038/s41598-021-95306-x

**Published:** 2021-08-09

**Authors:** Vegard Vinje, Erik N. T. P. Bakker, Marie E. Rognes

**Affiliations:** 1 Department of Scientific Computing and Numerical Analysis, Simula Research Laboratory, Martin Linges vei 25, 1364 Fornebu, Norway; 2 Department of Biomedical Engineering and Physics, Amsterdam University Medical Center, Meibergdreef 9, 1105 AZ Amsterdam, The Netherlands; 3 Department of Mathematics, University of Bergen, Bergen, Norway

**Keywords:** Computational models, Biophysical models, Applied mathematics

## Abstract

Fluid flow in perivascular spaces is recognized as a key component underlying brain transport and clearance. An important open question is how and to what extent differences in vessel type or geometry affect perivascular fluid flow and transport. Using computational modelling in both idealized and image-based geometries, we study and compare fluid flow and solute transport in pial (surface) periarterial and perivenous spaces. Our findings demonstrate that differences in geometry between arterial and venous pial perivascular spaces (PVSs) lead to higher net CSF flow, more rapid tracer transport and earlier arrival times of injected tracers in periarterial spaces compared to perivenous spaces. These findings can explain the experimentally observed rapid appearance of tracers around arteries, and the delayed appearance around veins without the need of a circulation through the parenchyma, but rather by direct transport along the PVSs.

## Introduction

Perivascular fluid flow of cerebrospinal or interstitial fluid in spaces surrounding brain blood vessels is recognized as a key component underlying brain transport and clearance^1–5^. In spite of its potential importance, several aspects of perivascular fluid flow are poorly characterized. In particular, the shape and structure of perivascular spaces (PVSs), as well as differences between periarterial and perivenous spaces and flow, remain enigmatic and disputed.

Traditionally, PVSs at the brain surface (pial PVSs) have been described and represented as annular structures surrounding the vasculature, either disjoint from the subarachnoid space (SAS)^3,6–8^, or as widenings of the SAS with reduced flow resistance^9^. When observing injected tracers in the murine brain via in-vivo two-photon imaging and fluorescence microscopy, the tracer is predominantly found along pial arteries and arterioles^7,10^. Careful examination of such images^11^ indicates that after an initial spread of tracer along arteries, the tracer is later present over the entire surface of the brain, thus suggesting tracer movement from the pial PVS into the adjacent SAS. The PVSs around arteries also appear to connect to the PVSs of veins, either via the SAS or directly where arteries and veins cross^11^. These observations correspond well with other postmortem confocal images in which the tracer signal in the PVSs of arteries, veins, and SAS were continuous^9^. In this study we will adopt the concept of PVSs on the brain surface as presented by Bedussi et al.^9^: the term PVS describes the fluid space surrounding blood vessels, continuous with the SAS and bounded by the arachnoid mater and the pia mater.

Perivascular fluid flow around arteries has now been studied extensively, while less evidence exists regarding the role and characteristics of perivenous flow. In and around large arteries on the brain surface, it seems well documented that the bulk fluid flow direction follows the blood flow direction^7,10^. Perivenous spaces have been suggested as exit routes, draining interstitial fluid and solutes out of the brain and the brain environment^12^. Evidence for this view includes later arrival of intracisternally injected tracer to venous PVSs compared to arterial PVSs, and tracer accumulation around venous PVSs after intraparenchymal injection^3^. However, in earlier work, it was suggested that the direction of PVS fluid flow varies in a more unpredictable manner^2^.

The anatomy, shape, and structure of perivascular spaces may vary by species, by location, and over time^5^. In particular, there may be persistent and systematic differences between the spaces surrounding pial arteries and veins^9^. A key open question is how and to what extent differences in shape affect PVS fluid flow and transport. Several theoretical and computational models have used the space between two concentric cylinders to model the PVS^13–17^. Mestre et al.^7^ have suggested, based on live tracer studies, that PVSs may be non-connecting, with two disjoint compartments forming on each side of pial arteries. Following these observations, Tithof et al.^18^ considered a confined PVS separated from the SAS, and found that an elliptic PVS was optimal for fluid transport given a constraint on the cross sectional area. An elliptical model with two disjoint PVSs was also assumed by Kedarasetti et al. to study perivascular pumping^19^. However, to the best of our knowledge, the physiological implications of differences in shape on periarterial and perivenous flow and solute transport have not been adequately characterized. Perivenous flow within the brain parenchyma has been studied in a 1D network model by Faghih and Sharp^20^, but models of the perivenous space at a level of detail comparable to the modeling studies mentioned above^13–16,19^ is lacking.

In this paper, using computational modelling in both idealized and image-based geometries at the mouse scale, we study and compare fluid flow and solute transport in pial (surface) periarterial and perivenous spaces. We first systematically address how PVS fluid velocities change with differences in arterial and venous PVS geometries. Next, we predict arrival differences of tracer in perivascular spaces based on differences in fluid velocities. Our findings demonstrate that fluid flow and solute transport is more rapid in spaces of periarterial shape when compared to spaces of perivenous shape, and that these geometrical differences alone predict a delayed tracer arrival in pial perivenous spaces.

## Results

**Figure 1:**
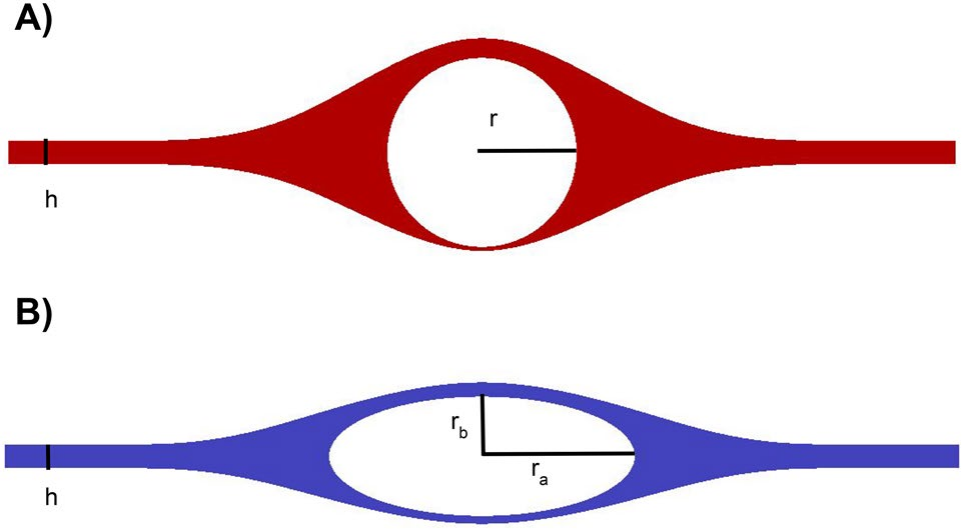
Computational domains for the idealized pial perivascular spaces surrounding an artery (**A**) and a vein (**B**) and simulating Stokes flow. In the baseline models, $r = 20\, \upmu \hbox {m}$, $r_a = 32\,\upmu \hbox {m}$, $r_b = 12.5\,\upmu \hbox {m}$ and $h = 5\,\upmu \hbox {m}$.

**Figure 2:**
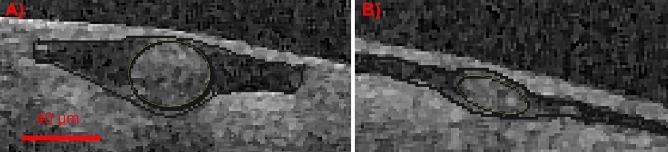
Blood vessels were imaged with optical coherence tomography (OCT). In vivo OCT images were taken from the surface of a human brain, after craniotomy. CSF appears as dark signal, and is present in the perivascular spaces (PVS) surrounding the blood vessels and the subarachnoid space (SAS). PVS and SAS appear to be continuous. These images were used to create image-based geometries for the artery (**A**) and the vein (**B**). Geometries were scaled to mouse size to be comparable to experimental data. Scale bar indicates dimensions after scaling.

**Figure 3:**
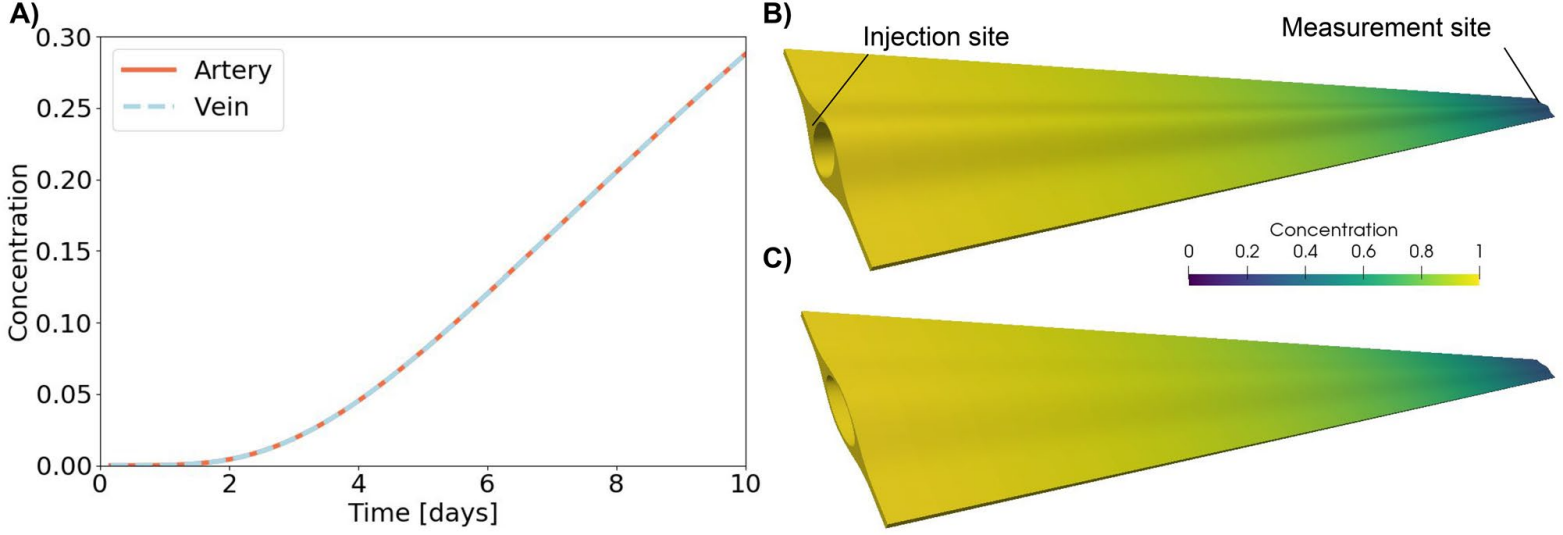
Diffusion of large (2000 kDa) tracers in PVS occurs on the time scale of days. In (**A**) the concentration of tracer at the point of measurement is shown for the artery and the vein. The two graphs are identical, showing that diffusion occurs at a similar rate in the two geometries. Tracer distribution after 10 days of diffusive transport is shown for the artery (**B**) and the vein (**C**).

Fluid flow and tracer transport were studied computationally in idealized (Fig. 1) and image-based (Fig. 2) geometries of arterial and venous PVSs (A0, V0, A1, and V1). The tracer concentration was set at a given injection site, and fluid flow was induced by a pressure difference along the length of the PVSs. The tracer then spread—by diffusion and/or convection—from the site of injection towards the site of measurement (see Fig. 3A,B). The rate of transport of tracer was dominated by convection and varied between different geometries.

### Diffusive transport is too slow to explain tracer movement along PVS

Even when measured only 5 mm from the injection site, diffusion was too slow to transport the large tracers of interest within the time frame (a few minutes) observed in experiments^7^: it took several days for an increase in tracer concentration to be detectable at the PVS measurement site (Fig. 3A). After 10 days, the average concentration at the measurement site was close to 30 % of the concentration at the injection site. Moreover, diffusion transported the tracer along the artery and along the vein at the same pace (Fig. 3). Figure 3B,C show the distribution along the artery (B) and the vein (C) after 10 days of diffusive transport. The tracer was evenly distributed at all cross-sections. Thus, for pure diffusion, the shape of the PVS did not influence the tracer transport.

### Periarterial geometries induce higher fluid velocities

**Figure 4:**
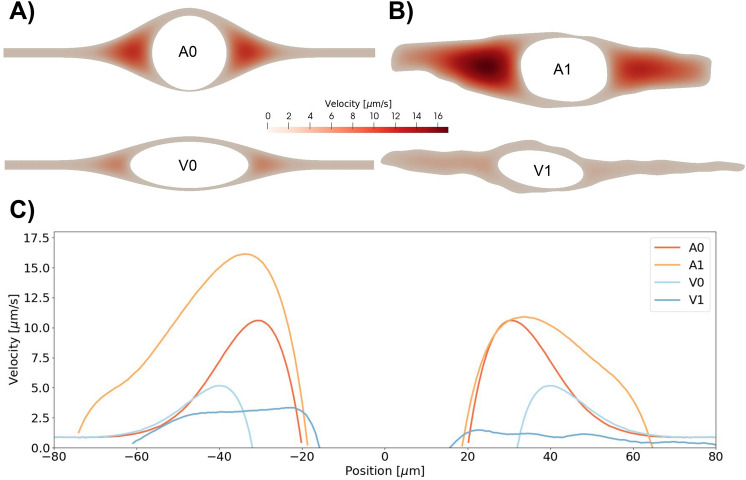
Velocity in the PVS around an artery and a vein in the idealized (**A**) and extracted (**B**) geometries. In these models, velocity has only one non-zero component, along the PVS. In both sets of geometries, flow is larger around arteries. In the idealized artery, PVS velocities reached 11 $\upmu$m/s in the largest open regions. In the idealized vein, fluid velocities reach 5 $\upmu$m/s. Flow between the pial surface and the vein as well as flow between the dura mater and the vein were negligible due to the small widths between these structures. In the image-based geometries PVS velocities reached 17 $\upmu$m/s around the artery and 3 $\upmu$m/s around the vein. (**C**) PVS velocity as a function of position along the horizontal centerline. $x = 0$ corresponds to the center of the vessel.

Fluid velocities and bulk flow in the PVS were induced by a static pressure gradient of 1.46 mmHg/m along the length of the PVS^16^. Although vascular pulsations play an important role in generating pulsatile flow in the PVS, the transport of solutes, as considered here, is dominated by bulk flow rather than pulsatile motion^21^. Velocities were higher in the larger open spaces surrounding the arteries (both idealized and image-based) than in other regions (see Fig. 4). The peak (and average) fluid velocity in the idealized periarterial and perivenous spaces was 11 (3) and 5 (1) $\upmu$m/s, respectively, and 17 (6) and 3 (1) $\upmu$m/s in the image-based periarterial and perivenous spaces. The velocity was higher on each side of the blood vessels, with little or no flow over the top or below the vessel. We note that even though the PVS forms an open space around the artery continuous with the SAS, the PVS flow pattern generated two distinct regions with higher fluid velocities. In the SAS far away from the vessel, the maximal fluid velocity was steady at $\approx$1 $\upmu$m/s in the idealized artery and vein models. Using the maximal fluid velocities *u* reported above, and the characteristic length *L* = 5 mm, the Peclet numbers Pe = $\frac{Lu}{D}$ ranged from Pe = 85000 (A1) to Pe = 110 (V1 with a 15-fold increase in diffusion coefficient).

With a pressure gradient of 0.01 mmHg/m, corresponding to CSF production alone (according to the third circulation^22^), the peak fluid velocity was 0.1 $\upmu$m/s in the idealized artery, approximately 100 times lower than in the other experiments and would not be able to explain rapid movement of tracer in the PVS (data not shown). In the geometry with the lowest convective velocity (the image-based perivenous space, V1), transport of 3 kDa Texas Red dextran was still dominated by convection. This 15-fold increase in diffusion coefficient accelerated early transport by approximately 3 min, and in particular the tracer concentration at the measurement site exceeded a threshold of 0.5 after 73 vs 76 min.

### Perivascular flow velocities vary linearly with PVS vascularity and width

**Figure 5:**
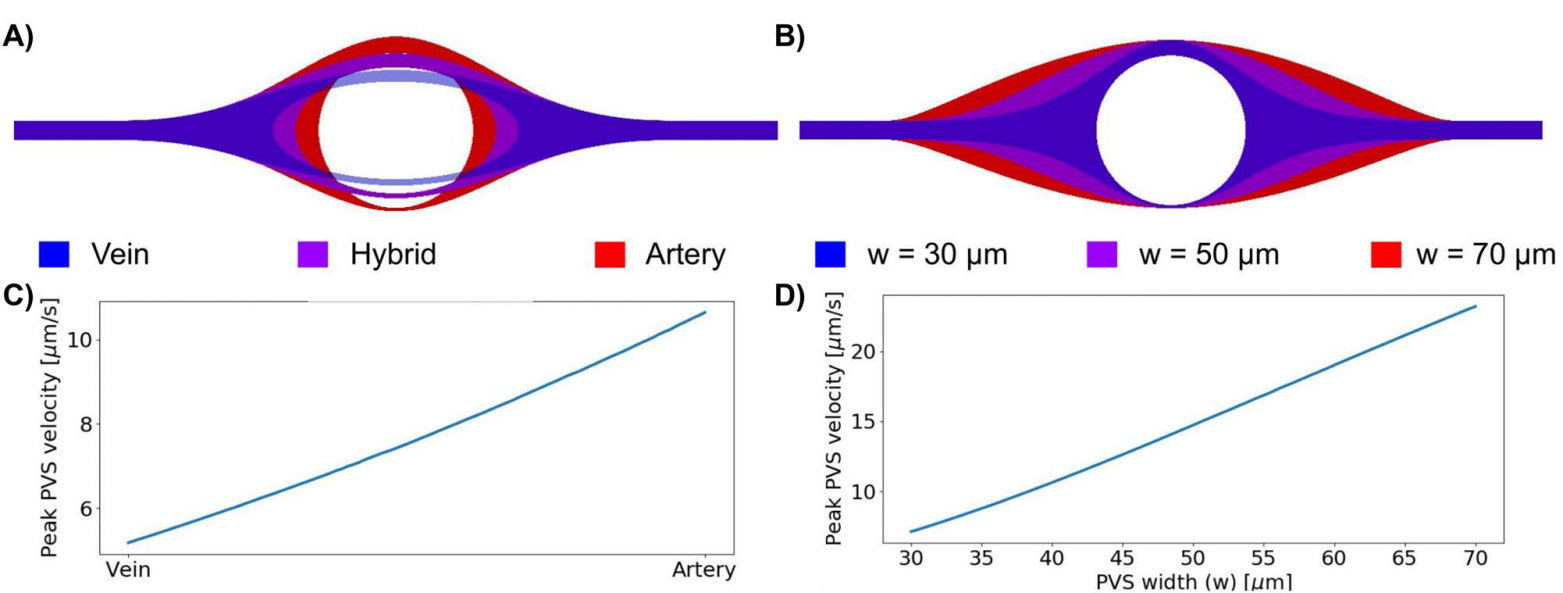
The effect of geometry change on maximal PVS velocity. (**A**) shows a gradual change from a venous to an arterial PVS. (**B**) shows change in PVS width for the arterial geometry. Maximal PVS velocities as a function of linear change in geometry is shown for the change from artery to vein (**C**), and changes in PVS width (**D**).In both cases the maximal PVS velocity increased linearly with changes in the geometry.

To determine the main geometrical parameters affecting the perivascular flow, we examined the peak perivascular fluid velocity resulting from a gradual change from the idealized periarterial geometry to the idealized perivenous geometry, and from changing PVS widths (see “Methods”). Linear transitions between these parameterized geometries resulted in near linear changes in peak perivascular flow velocities (Fig. 5, Additional files (videos) 1–2. An increase in PVS width from 40 $\upmu$m (default periarterial geometry) to 70 $\upmu$m increased PVS velocities from 11 (3) $\upmu$m/s to 23 (9) $\upmu$m/s, while a reduction to 30 $\upmu$m PVS width resulted in PVS velocities of 7 (2) $\upmu$m/s. Thus, a narrow periarterial space generated similar perivascular flow velocities as the idealized perivenous space (7 (2) $\upmu$m/s vs 5 (1) $\upmu$m/s). We note that the linear increase observed is in contrast to the quadratic relationship between the radius and maximal flow velocity in a cylinder.

### Tracer appears first around arteries, then around veins

**Figure 6:**
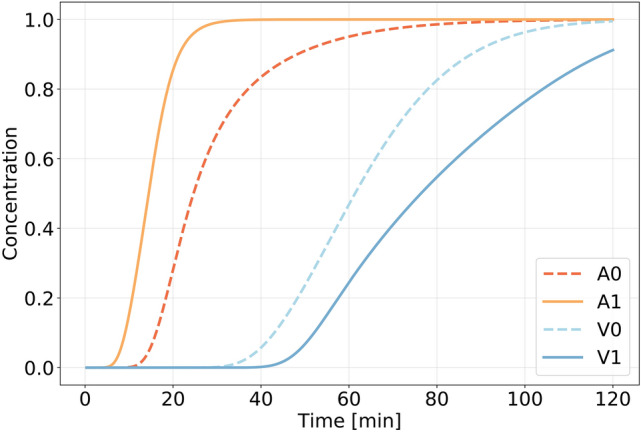
Time plots of average concentrations at the point of measurement in the idealized artery (A0) and vein PVS (V0) together with the average concentrations in the image-based artery (A1) and vein (V1) PVS. The difference between arterial and venous PVS transport is larger in the image-based geometries, but there is a significant delay in venous PVS compared to arterial PVS in both cases.

Simulations of convective and diffusive transport (via Eq. (3) in “Methods”) in the perivascular spaces revealed substantial differences between the periarterial and perivenous transport characteristics and the tracer arrival times at the measurement site (Fig. 6). In the periarterial spaces, the average tracer concentration at the measurement site rapidly grew after 5–20 min, with the most rapid transport and time to peak concentration ($\approx$25 min) in the image-based periarterial spaces. In the perivenous spaces, the measured concentration remained low until after 30–45 min before increasing from 35–45 min and peaking after more than 1.5 h. These differences are directly attributable to the differences in PVS velocities and geometries: in the idealized models, the peak and average periarterial fluid velocities were approximately twice those of the vein, and the convective front thus appears twice as fast around the artery. The difference between the artery and the vein was greater in the image-based models, due to faster transport along the artery and slower transport along the vein.

### Tracer patterns depend on time of measurement

**Figure 7:**
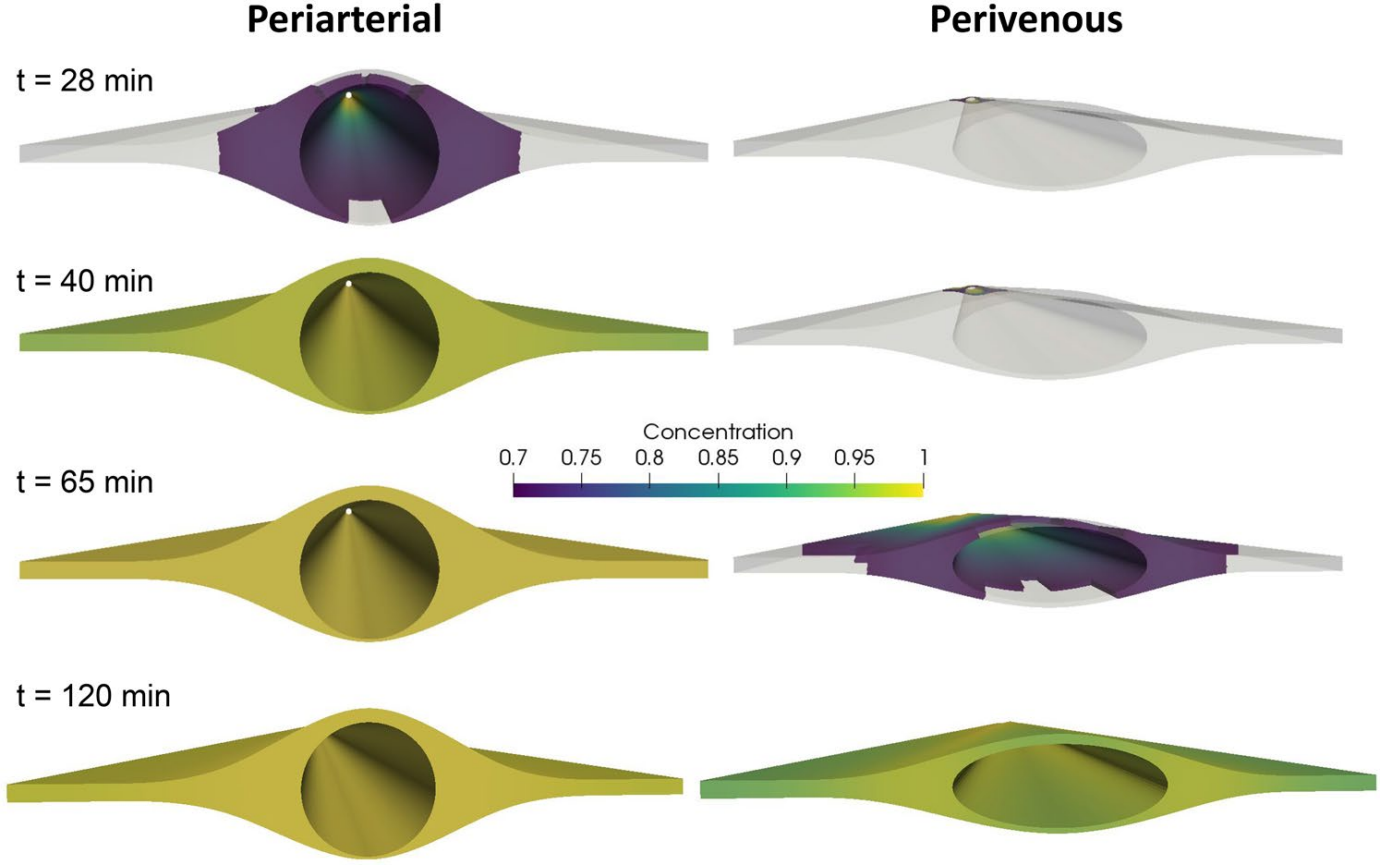
Tracer (2000 kDa) distribution around the artery (left) and the vein (right) at several time points after injection in the idealized geometries. The front end shows the site of measurement, while the point of injection is in the far end of the geometry. Colors indicate a threshold of 0.7. At 28 min after injection, the areas of intense concentration around the artery suggest two disjoint PVS. After 40 min, the concentration is almost uniform at the cross section at the artery PVS, while no tracer is found around the vein. At 65 min after injection, tracer appears around the vein, apparently forming two disjoint PVS. After 120 min, tracer is distributed almost uniformly over both cross sections.

**Figure 8:**
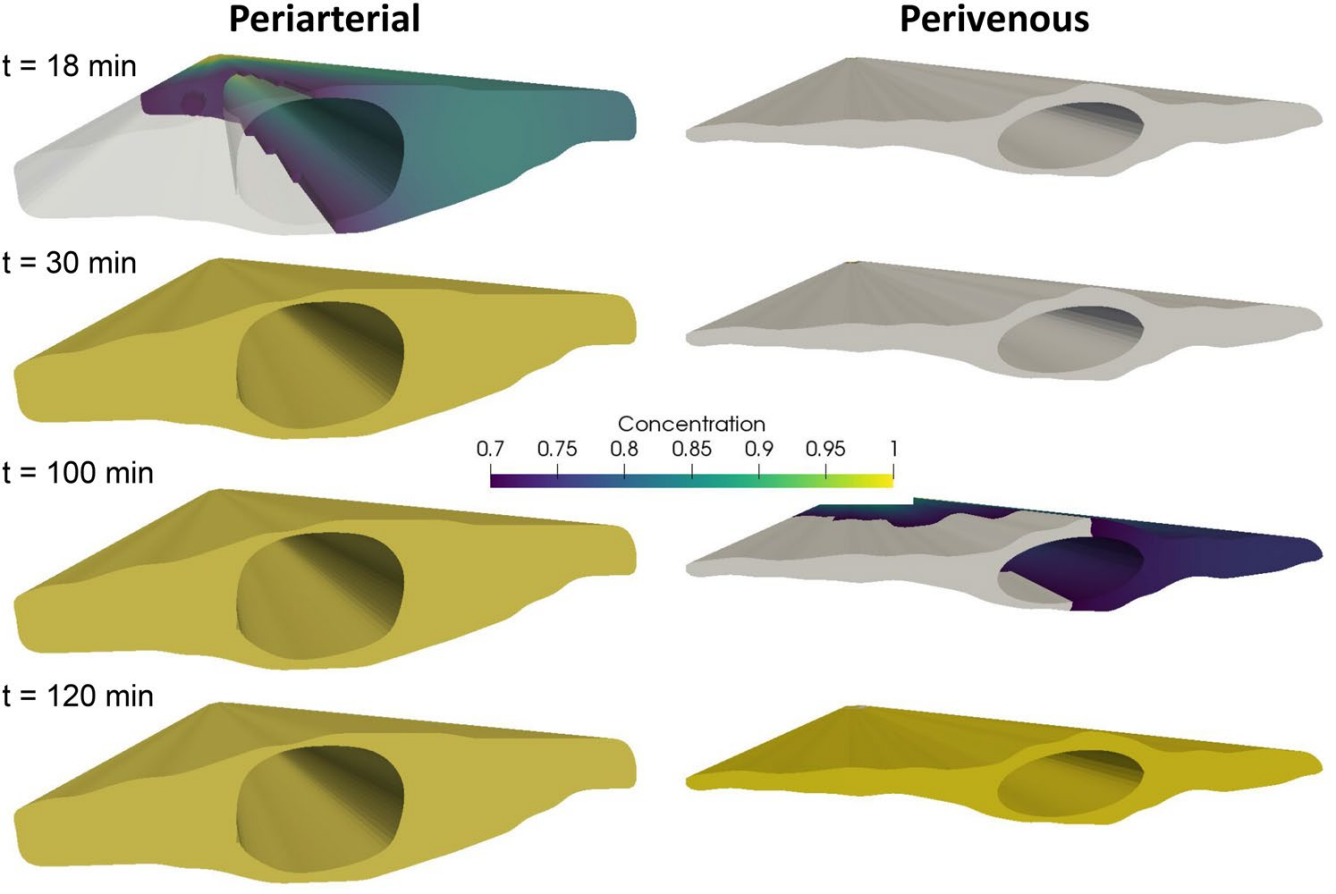
Tracer (2000 kDa) distribution around the artery (left) and the vein (right) at several time points after injection. The front end shows the site of measurement, while the point of injection is in the far end of the geometry. Colors indicate a threshold of 0.7. At 18 min after injection, tracer appears only on the right side of the artery, while after 30 min the concentration in the periarterial space is evenly distributed. After 100 min, the concentration is almost uniform at the cross section at the artery PVS, while the venous PVS is half covered, despite the fact that there is a small path for communication between the PVS on each side of the vessel. At 120 min after injection, tracer distributes almost uniformly around both the artery and the vein. In the extracted geometries we note asymmetry due to slightly larger PVS on one side of the vessel.

In Figs. 7 and 8, we visualize the concentration field (above a given threshold) at the PVS measurement sites at a series of time points (see also Additional files (videos) 3–4). After around 20 min, tracer had started to appear around the artery in the idealized model (Fig. 7, $t =$ 28 min). The corresponding apparent structure of the periarterial space is two disjoint compartments, one on each side of the artery. However, after 40 min, the tracer was distributed in the entire PVS cross-section. Analogously, 65 min after injection tracers appeared around the idealized vein and also seem to reveal two disjoint avenues of transport. After 100 min, both the arterial and venous PVSs were filled at the cross-sectional area at the measurement site in the idealized models. For the image-based models, the trends were similar, but included asymmetry due to the asymmetric geometries (Fig. 8). After 15–20 minutes, tracer appeared on both sides of the image-based periarterial space, while it took up to 90–100 min for the venous PVS to reach similar levels of concentration at the measurement site.

## Discussion

In this study, we used computational fluid dynamics to investigate differences in tracer transport along surface vessels of the brain. The geometrical models involved both idealized and image-based vessels. In both cases, we found higher PVS velocities in periarterial spaces compared to perivenous spaces. These differences were caused by the geometrical differences between the spaces and resulted in much faster transport along arteries than along veins. The surface vessels and PVSs modeled in this study should be distinguished from penetrating vessels and capillaries which may have different surroundings^23,24^.

In agreement with previous studies^15,25^, we show that for large molecules, such as e.g. 2000 kDa dextran, pure diffusion is too slow to explain the rapid tracer transport observed along the brain surface^7,10^. Diffusive transport of the large tracers studied here occurs on the scale of days and weeks and not minutes or hours. Dispersion induced by arterial pulsations has been proposed as a mechanism to explain solute transport even in the absence of bulk flow^15,25^. However, an increase by a factor of approximately two as demonstrated by Asgari et al.^15^ does not seem sufficient to account for the rapid transport of large tracers in pial PVSs^7,10^. By analytical models, Troyetsky et al.^21^ have reached the same conclusion, namely that bulk flow velocities of $\approx$20 $\upmu$m/s are much more effective in transporting tracers than dispersion by arterial pulsations. On the other hand, within the brain parenchyma, transport of smaller tracers (e.g. <1 kDa gadubutrol) occur but slightly faster than by diffusion alone^26–28^, and within a similar order of magnitude.

In our study, periarterial fluid velocities were but slightly lower than experimental evidence suggests (peak velocities of 11-23 $\upmu$m/s compared to $\approx$10-40 $\upmu$m/s)^7,10^. Pulsatile flow patterns, not included in our models here, could likely enhance tracer transport further, but the net transport induced by the unidirectional bulk flow considered here would dominate that effect. According to data presented by Iliff et al.^11^, reduced vessel pulsations reduce tracer intensity at the measurement site by a factor close to 2. Local arterial wall pulsations can induce a small net flow, of 0.1 $\upmu$m/s over a 5 mm long PVS or up to 7 $\upmu$m/s in very long (100 mm) PVSs^16^. On the other hand, tracer infusions increase the intracranial pressure (ICP)^11^, and may also give rise to a small but long-lasting ICP gradient^29^. Yet, the experimentally observed rapid flows of macroparticles and large tracers in the pial PVSs do not seem to hinge upon such ICP increases^30^. A small static pressure gradient seems to be necessary for net flow of the reported magnitude^16,19^, yet the required gradient is of similar magnitude as naturally pulsatile pressure gradients within the brain^31^.

In our simulations, tracers appeared first around arteries (after 5–20 min), then around veins (1–2 h). These observations can be ascribed to the fact that PVS velocities were higher in the vicinity of arteries than around veins. According to the original glymphatic hypothesis^3^, CSF flows in along arteries, CSF/ISF flows through the interstitium, and ISF is cleared along paravenous routes. The later appearance of tracers around veins on the brain surface would then be explained by this recirculation and clearance pathway. However, the delay observed by Iliff et al.^3^ (0–30 min vs 1–2 h for arteries vs veins) compares well with the results from our simulations for which no such recirculation was assumed. Instead, our data suggest that differences in periarterial and perivenous geometries explain the later appearance of tracer around veins, and not necessarily a full recirculation through a (glymphatic) interstitial pathway. We also note that, in agreement with our hypothesis, Ma et al.^32^ observed spread of tracer directly from branches of the middle cerebral artery to some of the larger veins. In their study, it was concluded that tracer spread over the dorsal hemisphere was confined both to larger caliber arteries and veins at the brain surface^32^. In light of these views^3,32^, we conjecture that increased PVS velocities would increase the tracer clearance rate from the intracranial compartment. In the glymphatic theory^3^, increased PVS velocities could also be associated with more rapid transport into the brain parenchyma.

Weller and coauthors^33^ and many subsequent studies^8,13–17,19,34^ have suggested or assumed that pial PVSs take the form of annular or elliptic tubes that are separate(d) from the SAS, while Nedergaard et al.^7,18^ also suggest that the PVS forms disjoint compartments on each side of the blood vessels. We show here that thresholded representations of tracer concentration, and thus likely volumetric fluorescence images, will highlight areas with high bulk flow and essentially hide areas with low bulk flow if the measurement site is some distance away from the site of injection. More precisely, tracer images can, at some time points, display PVSs as separate both from the SAS and each other when they in fact form one continuous space (Figs. 7, 8) The effect of this gap on bulk flow may not be large, especially if the gap connecting two PVS around a vessel is small, but allows for diffusion and mixing of particles between the two spaces.

Imaging of the brain surface of humans and mice with OCT suggests that the PVS are widened sections of the SAS^10^. The round shape of arteries, which results from the relatively high intravascular pressure, may physically open up the space between the arachnoid and pia mater. Further away from vessels, the SAS may nearly collapse and not even be detectable. This could create the impression that PVS are separated from the SAS. From volumetric imaging of tracer transported via bulk flow, we show that the distinction between the two views might not be obvious. In both cases, more tracer will be seen in the large PVS areas on each side of the vessel, while there will be less, or even no tracer at all in other areas. In particular, our results demonstrate that tracer accumulation around vessels is not in disagreement with the theory that the PVS and SAS form one continuous space. In data provided by Schain et al.^8^, the spread of tracer along PVS occurs rapidly, and travels $\approx$1 mm in a few minutes. Transport along the cortex surface perpendicular to the PVS however is much slower and only covers a few $\upmu$m during the same time period, a transport more in line with diffusion given the size of tracer (3 kDa) used in their experiments^8^).

In terms of limitations of our study, we have not included arterial pulsations as a driving mechanism for transport. This is a limitation that needs clarification as pulsations have been proposed to be the main driver of pulsatile PVS flow^11,30^ and even net flow^7,13^. However, as recently pointed out by Troyetsky et al.^21^, the bulk flow, and not the oscillatory movement, is the main mechanism for tracer transport. As our simulated net flow was close to the experimentally observed range, we would expect to see only a minor additional effect of vessel wall pulsations or pulsatile pressure gradients on tracer transport. As assessment of tracer transport, and not the mechanism behind net flow, is the purpose of this study, this is a valid simplification. Moreover, the pressure gradient imposed was assumed equal for the periarterial and perivenous spaces. If pulsatile motion plays a significant role in driving perivascular fluid flow, our simulations would overestimate tracer transport along perivenous spaces as they have lower pulsatility. However, if this effect exists and was included, the difference in tracer transport between arteries and veins would increase, not decrease, and our conclusion remain the same. Similarly, by including brain compliance and fluid or solute transport across the pial membrane, one could imagine a minor additional change in fluid velocities and dispersion^35^.

Throughout, we have discussed our results in the context of and based on experimental data from mice. We note however, that the OCT images used to generate the image-based geometries were taken from a human subject (and then correctly scaled). This choice was based on the relatively poor image resolution compared to the size of a mouse PVS. We also note that differences in structure between mouse and human PVS and/or SAS may exist. We further note that we (by image quality) selected only two vessels to generate the image-based domains although we expect there to be large differences within both types of vessels (arteries and veins). The two extracted vessels were of different size, which may impact the size of the adjacent PVS. Yet, our findings from these image-based and (over 200) idealized geometries show that any consistent difference in shape between arterial and venous PVS will lead to a change in transport velocity, and consequently a different time of appearance of tracer around arteries versus veins. The images were extracted in 2D and extruded in the z-direction, and the PVS velocity was thus computed independent of the z-coordinate (velocity distribution is assumed equal in all cross-sections of the PVS). We thus omit bifurcations and the curvature of the vessel. However, as PVS flow has a very low Reynolds number (Re < 0.01), and as we have previously shown that bifurcations do not introduce complex flow patterns in the PVS^16^, we consider this simplification to be reasonably valid. The concentration threshold used to render volumetric images was arbitrarily set; a change in this value would not alter our conclusions, but would shift the arrival time of tracer shown in Figs. 7 and 8.

In conclusion, we have shown that geometry differences between arterial and venous pial PVSs may lead to higher net CSF flow, more rapid tracer transport and earlier arrival times of injected tracers in periarterial spaces compared to perivenous spaces. These findings are in agreement with experimentally observed differences in tracer arrival times, and may explain the rapid appearance of tracers around arteries, and the delayed appearance around veins without the need of a circulation through the parenchyma, but rather by direct transport along the PVS.

## Methods

### Domains and meshes

We studied families of three-dimensional geometries $\Omega$ of pial PVSs: (a) families of idealized PVSs around an artery (A0) and/or a vein (V0), and (b) an image-based set of PVSs surrounding an artery (A1) and/or a vein (V1) extracted from human optical coherence tomography (OCT) images. To compare with experimental data of tracer transport, all vessels and PVSs were scaled to the mouse size (such that $r = 20\,\upmu \hbox {m}$ for the arteries in agreement with^7^). Cross-sections of the periarterial and perivenous spaces are illustrated in Figs. 1 (idealized) and 2 (image-based); these cross-sections were extruded by 5 mm along the third axis to define the computational domains.

Each cross-section of the idealized periarterial spaces was assumed to surround a circular artery and to be continuous with the SAS with more open space on each side of the vessel than above and below^9^. The outer PVS surfaces were defined by spline curves via sets of control points (for .geo-files, see the data set^36^). The minimal SAS height (h) was set to 5 $\upmu$m, the maximal SAS height (including the artery with diameter 2r = 40 $\upmu$m) was set to 45 $\upmu$m, and the PVS width representing the distance between the arterial surface and the end of the PVS tapering was set to 40 $\upmu$m. For the idealized perivenous spaces, the vein was assumed to form an ellipse with major axis $r_a =$ 32 $\upmu$m and minor axis $r_b =$ 12.5 $\upmu$m. The cross-sectional area of the artery and the vein are thus equal. In the narrow SAS region, the perivenous height was comparable to that of the artery (h), while the maximal SAS height (including the vein) was 30 $\upmu$m.

To systematically quantify the effect of PVS geometry on maximal PVS flow velocity, we created two further families of computational domains parametrized by (i) a vascularity $\rho \in [0, 1]$ (with $\rho = 0$ and $\rho = 1$ corresponding to the venous and arterial PVS, respectively, and $\rho \in (0, 1)$ linearly transforming between these two geometries) and (ii) the periarterial width $w \in (30, 70)\,\upmu \hbox {m}$. We note that the term width here reflects an extension of the PVS mainly in the horizontal direction (see Fig. 5B), and thus does not represent an overall increase in PVS radius in all directions. For both families, we uniformly sampled 100 meshes. The gradual changes in geometry for the two cases are visualized in Fig. 5 and Additional Files (Videos) 1–2.

The image-based geometries were constructed from previously published OCT data(^10^, see below for a brief description) in which the PVSs surrounding two vessels were easily recognizable and identifiable, and meshed via Gmsh^37^.

### OCT imaging

Images of human and mouse paravascular spaces (mice OCT data shown elsewhere [10]) were collected using Optical Coherence Tomography (OCT). Human OCT data (n=5) were recorded using a commercial 50 kHz Santec IVS 2000 swept source OCT system operating at a centre wavelength of 1300 nm. The full width at half maximum (FWHM) axial and lateral resolutions were measured at 14 $\upmu$m and 25$\upmu$m, respectively. Volumetric images (x,y,z) of 10 mm by 10 mm by 4 mm, containing 1024 by 1024 by 600 pixels were collected from healthy brain tissue before tumor surgery, after craniotomy and the removal of the dura. The clinical procedures were carried out at the Radboud University (Nijmegen, the Netherlands) under approval by the local medical ethical committee of the Radboud University in accordance with the Declaration of Helsinki for experiments involving humans. From each patient, we obtained written informed consent. Privacy of the patients was ensured by anonymization. The collected OCT images were post-processed to enhance image quality. Using ImageJ software, two adjacent B scans were averaged to reduce the speckle prior to applying two filters (i.e. despeckle and sharpen) and enhancing the contrast. The depth axis of the OCT images was corrected with a refractive index of 1.3, whereafter the pixels were scaled to isotropic dimensions.

### Computational fluid dynamics in the pial PVS

In the pulsatile perivascular flow observed experimentally^7,10^, flow amplitudes are similar compared to the net flow ($\approx$10 $\upmu$m/s vs $\approx$20 $\upmu$m/s). The dominant transport mechanism over time will thus be the net movement of fluid, with only minor effects of mixing^21^. Therefore, together with the assumptions that pial PVSs are continuous with the SAS^9^ and open^38^, we modelled the convective flow in the PVS as an incompressible Stokes flow; that is, the fluid velocity $v = v (x, y, z)$ and pressure $p = p(x, y, z)$ for $(x, y, z) \in \Omega$ solve

1a $$\begin{aligned} \mu \nabla ^2 v - \nabla p&= 0, \end{aligned}$$

1b $$\begin{aligned} \nabla \cdot v&= 0, \end{aligned}$$

under additional given boundary conditions.

In the extruded domains considered here, in which each cross-section of each geometry is constant, the incompressible Stokes equations subject to a constant pressure difference over the domain length can be reduced to the following equation for the axial velocity $v = v(x, y)$ (velocity in the z-direction) over the two-dimensional cross-sections:

2 $$\begin{aligned} \mu \nabla ^2 v - \frac{\partial p}{\partial z} = 0. \end{aligned}$$

We let the flow be driven by a constant pressure gradient $\tfrac{\partial p}{\partial z} = 1.46$ mmHg/m. Even though such a gradient has only been reported as a pulsatile gradient^31^, static gradients of this magnitude have been shown to create net velocities of around 20 $\upmu$m/s in the PVS^16^ in agreement with experimental observations of injected microspheres in pial PVS. We also conducted experiments with a lower pressure gradient: $\tfrac{\partial p}{\partial z} = 0.01$ mmHg/m^31^ corresponding to the third circulation’s production of CSF alone^22^. As the third circulation, we here refer to a constant production of CSF from the choroid plexus within the ventricles and slow flow through the aqueduct, into the SAS and eventually absorption of CSF at the arachnoid granulations. On the vessel wall, and on the upper (arachnoid mater) and lower (pia mater) walls we assumed a no slip condition (i.e. $v = 0$), while on the side walls (extending further in the SAS), we used the natural Neumann (symmetry) condition ($\mu \frac{\partial u}{\partial n} = 0$).

### Convection–diffusion modelling

We simulated the distribution and evolution of a solute (e.g. tracer or microspheres) in the pial PVS in response to a steady injection at one end ($z = 0$) of the PVS geometries. We assume that the solute concentration $u = u(x, y, z, t) \in \Omega$ will be governed by the convection–diffusion system:

3a $$\begin{aligned} \frac{\partial u}{\partial t} + v \cdot \nabla u&= D\nabla ^2 u \quad \text { in } \Omega , \end{aligned}$$

3b $$\begin{aligned} u&= 1 \quad \text { on } \partial \Omega _\text {in}, \end{aligned}$$

3c $$\begin{aligned} D\nabla u \cdot n&= 0 \quad \text { on } \partial \Omega _e, \end{aligned}$$

3d $$\begin{aligned} u&= 0 \quad \text { at } t = 0, \end{aligned}$$

where *v* is the convective fluid velocity, and *D* is the diffusion coefficient in water for the given solute. We denote the inlet end of the geometry by $\partial \Omega _\text {in}$, while $\partial \Omega _e$ is the remaining part of the domain boundary $\partial \Omega$. The boundary and initial conditions (3b)–(3d) describe a constant injection at one end of the PVS geometry with no initial tracer anywhere in the PVS or SAS. To model pure diffusion in the PVS, we set $v = 0$. When including convection, we first solved the pressure-driven Stokes Equation (2) to find the fluid velocity $v = v(x, y) = v(x,y,z)$.

### Material parameters

We set the CSF viscosity $\mu$ = 0.7 Pa s^16^. The diffusion coefficient *D* varies by molecular size, and small particles typically diffuse faster than larger particles. In this study we set the diffusion coefficient to $D = 6.8 \times 10^{-8}$ cm$^2$/s^39^ to represent unhindered diffusion in the CSF-filled PVS of the large (2000 kDa) tracer used for volumetric imaging by Mestre et al^7^. Microspheres of approximately 1 $\upmu$m in diameter^7^ are much larger and would thus be expected to diffuse even slower. In addition to the small diffusion coefficient for the 2000 kDa tracer, we performed simulations using the $\approx$15x higher diffusion coefficient $D = 113 \times 10^{-8}$ cm$^2$/s^39^ of 3 kDa Texas Red dextran as well.

### Quantities of interest

The concentration *u* is a normalized value (between 0 and 1) and set to 1 at the injection site ($z = 0$). As our primary quantity of interest, we measure the average tracer concentration $\bar{u}$, averaged over the cross-section $\partial \Omega _{\mathrm{end}}$ at the end of the domain (where $z = L$), over time:

4 $$\begin{aligned} \bar{u}(t) = \tfrac{1}{A} \int _{\partial \Omega _{\mathrm{end}}} u \, \mathrm {d}x, \end{aligned}$$

where *A* is the area of $\partial \Omega _{\mathrm{end}}$. In addition to $\bar{u}(t)$, we report the velocity and concentration fields in the periarterial and perivenous spaces.

### Numerical solution and verification

The partial differential equations were solved using the finite element method and the FEniCS software suite^40^. The reduced Stokes equations (2) were solved using a continuous piecewise linear approximation (in space) giving second order accurate approximation of the axial velocity. The convection–diffusion equations () were solved using a continuous piecewise linear finite element approximation in space and an implicit Euler discretization in time with a uniform time step $\Delta t =$ 10 s and mesh resolution $\Delta x =$ 1 $\upmu$m until $T = 120$ min. The discrete velocity field $v = v(x, y)$ was interpolated onto each vertex of the three-dimensional mesh: $v(x, y, z) = v(x, y)$. For the pure diffusion simulations, we used a time step of $\Delta t =$ 1 day and an end time of $T = 150$ days. To validate our CFD solver, we simulated pressure driven flow in concentric cylinders with $r_0 = 20\,\upmu \hbox {m}$ and $r_1 = 60\,\upmu \hbox {m}$. We used the same pressure gradient and viscosity as in our simulation setup. The simulated fluid velocity was compared with the exact solution for the problem^41^, and the comparison is shown in Additional File 5. The relative error in terms of maximal velocity was found to be 0.012 for a mesh size that was coarser than those presented in the results section (Additional File 5). For the simulations presented in the results section, numerical convergence tests were performed to ensure that the reported results were converged, independent of mesh resolution and the choice of time step (see Additional File 6). The resolution of the mesh used for simulations are shown in Additional File 7.

## Supplementary Information

Supplementary Information 1.

Supplementary Information 2.

Supplementary Information 3.

Supplementary Information 4.

Supplementary Information 5.

Supplementary Information 6.

Supplementary Information 7.

Supplementary Legends.

## Data Availability

The mesh-files used in this study can be found at the repository^36^. Additional files can be provided upon request to the corresponding author.

## References

[1] Rennels ML, Gregory TF, Blaumanis OR, Fujimoto K, Grady PA (1985). Evidence for a paravascular fluid circulation in the mammalian central nervous system, provided by the rapid distribution of tracer protein throughout the brain from the subarachnoid space. Brain Res..

[2] Ichimura T, Fraser P, Cserr HF (1991). Distribution of extracellular tracers in perivascular spaces of the rat brain. Brain Res..

[3] Iliff JJ (2012). A paravascular pathway facilitates CSF flow through the brain parenchyma and the clearance of interstitial solutes, including amyloid $$\beta$$. Sci. Transl. Med..

[4] Bakker EN (2016). Lymphatic clearance of the brain: Perivascular, paravascular and significance for neurodegenerative diseases. Cell. Mol. Neurobiol..

[5] Troili F (2020). Perivascular unit: This must be the place. The anatomical crossroad between the immune, vascular and nervous system. Front. Neuroanat..

[6] Weller R, Subash M, Preston S, Mazanti I, Carare R (2008). Perivascular drainage of amyloid-$$\beta$$ peptides from the brain and its failure in cerebral amyloid angiopathy and Alzheimers disease. Brain Pathol..

[7] Mestre H (2018). Flow of cerebrospinal fluid is driven by arterial pulsations and is reduced in hypertension. Nat. Commun..

[8] Schain AJ, Melo-Carrillo A, Strassman AM, Burstein R (2017). Cortical spreading depression closes paravascular space and impairs glymphatic flow: Implications for migraine headache. J. Neurosci..

[9] Bedussi B (2017). Paravascular channels, cisterns, and the subarachnoid space in the rat brain: A single compartment with preferential pathways. J. Cereb. Blood Flow Metab..

[10] Bedussi B, Almasian M, de Vos J, VanBavel E, Bakker EN (2018). Paravascular spaces at the brain surface: Low resistance pathways for cerebrospinal fluid flow. J. Cereb. Blood Flow Metab..

[11] Iliff JJ (2013). Cerebral arterial pulsation drives paravascular CSF-interstitial fluid exchange in the murine brain. J. Neurosci..

[12] Jessen NA, Munk ASF, Lundgaard I, Nedergaard M (2015). The glymphatic system: A beginners guide. Neurochem. Res..

[13] Wang P, Olbricht WL (2011). Fluid mechanics in the perivascular space. J. Theor. Biol..

[14] Sharp MK, Carare RO, Martin BA (2019). Dispersion in porous media in oscillatory flow between flat plates: Applications to intrathecal, periarterial and paraarterial solute transport in the central nervous system. Fluids Barriers CNS.

[15] Asgari M, De Zélicourt D, Kurtcuoglu V (2016). Glymphatic solute transport does not require bulk flow. Sci. Rep..

[16] Daversin-Catty C, Vinje V, Mardal KA, Rognes ME (2019). The mechanisms behind perivascular fluid flow. Plos one.

[17] Romanò F, Suresh V, Galie PA, Grotberg JB (2020). Peristaltic flow in the glymphatic system. Sci. Rep..

[18] Tithof J, Kelley DH, Mestre H, Nedergaard M, Thomas JH (2019). Hydraulic resistance of periarterial spaces in the brain. Fluids Barriers CNS.

[19] Kedarasetti RT, Drew PJ, Costanzo F (2020). Arterial pulsations drive oscillatory flow of CSF but not directional pumping. Sci. Rep..

[20] Faghih MM, Sharp MK (2018). Is bulk flow plausible in perivascular, paravascular and paravenous channels?. Fluids Barriers CNS.

[21] Troyetsky DE, Tithof J, Thomas JH (2021). Dispersion as a waste-clearance mechanism in flow through penetrating perivascular spaces in the brain. Sci. Rep..

[22] Cushing H (1925). The third circulation and its channels. Lancet.

[23] Pizzo ME (2018). Intrathecal antibody distribution in the rat brain: Surface diffusion, perivascular transport and osmotic enhancement of delivery. J. Physiol..

[24] Hannocks M-J (2018). Molecular characterization of perivascular drainage pathways in the murine brain. J. Cereb. Blood Flow Metab..

[25] Rey J, Sarntinoranont M (2018). Pulsatile flow drivers in brain parenchyma and perivascular spaces: A resistance network model study. Fluids Barriers CNS.

[26] Croci M, Vinje V, Rognes ME (2019). Uncertainty quantification of parenchymal tracer distribution using random diffusion and convective velocity fields. Fluids Barriers CNS.

[27] Valnes LM (2020). Apparent diffusion coefficient estimates based on 24 hours tracer movement support glymphatic transport in human cerebral cortex. Sci. Rep..

[28] Ray L, Iliff JJ, Heys JJ (2019). Analysis of convective and diffusive transport in the brain interstitium. Fluids Barriers CNS.

[29] Vinje V, Eklund A, Mardal K-A, Rognes ME, Støverud K-H (2020). Intracranial pressure elevation alters CSF clearance pathways. Fluids Barriers CNS.

[30] Raghunandan A (2021). Bulk flow of cerebrospinal fluid observed in periarterial spaces is not an artifact of injection. Elife.

[31] Vinje V (2019). Respiratory influence on cerebrospinal fluid flow—A computational study based on long-term intracranial pressure measurements. Sci. Rep..

[32] Ma Q (2019). Rapid lymphatic efflux limits cerebrospinal fluid flow to the brain. Acta Neuropathol..

[33] Zhang E, Inman C, Weller R (1990). Interrelationships of the pia mater and the perivascular (virchow-robin) spaces in the human cerebrum. J. Anat..

[34] Bilston LE, Stoodley MA, Fletcher DF (2010). The influence of the relative timing of arterial and subarachnoid space pulse waves on spinal perivascular cerebrospinal fluid flow as a possible factor in syrinx development: Laboratory investigation. J. Neurosurg..

[35] Kedarasetti RT (2020). Functional hyperemia drives fluid exchange in the paravascular space. Fluids Barriers CNS.

[36] Vinje V, Bakker EN, Rognes ME (2021). Mesh files for idealized and image-based periarterial and perivenous spaces. bioRxiv.

[37] Geuzaine C, Remacle J-F (2009). Gmsh: A 3-d finite element mesh generator with built-in pre-and post-processing facilities. Int. J. Numer. Methods Eng..

[38] Min Rivas F (2020). Surface periarterial spaces of the mouse brain are open, not porous. J. R. Soc. Interface.

[39] Sandrin D (2016). Diffusion of macromolecules in a polymer hydrogel: From microscopic to macroscopic scales. Phys. Chem. Chem. Phys..

[40] Alnæs M (2015). The fenics project version 1.5. Arch. Numer. Softw..

[41] Mott J, Joseph D (1968). Stability of parallel flow between concentric cylinders. Phys. Fluids.

