# Supplementary figures and images for: Brain solute transport is more rapid in periarterial than perivenous spaces
Source: Sci Rep. 2021 Aug 9;11:16085. doi: 10.1038/s41598-021-95306-x (PMC8352970; doi:10.1038/s41598-021-95306-x)

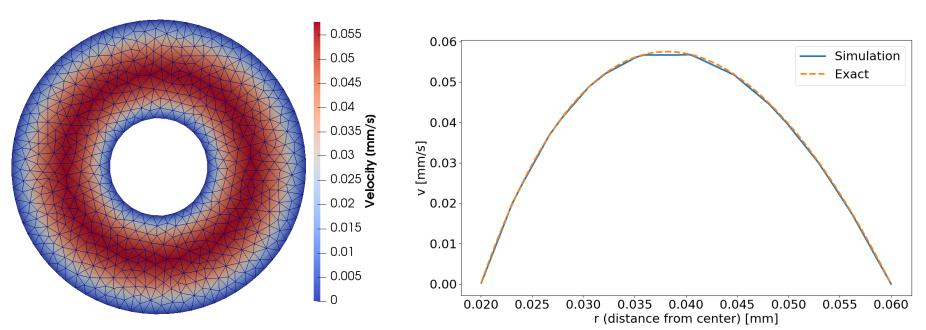

Supplement: Supplementary file 5 — Supplementary Information 5. [file 41598_2021_95306_MOESM5_ESM.png]

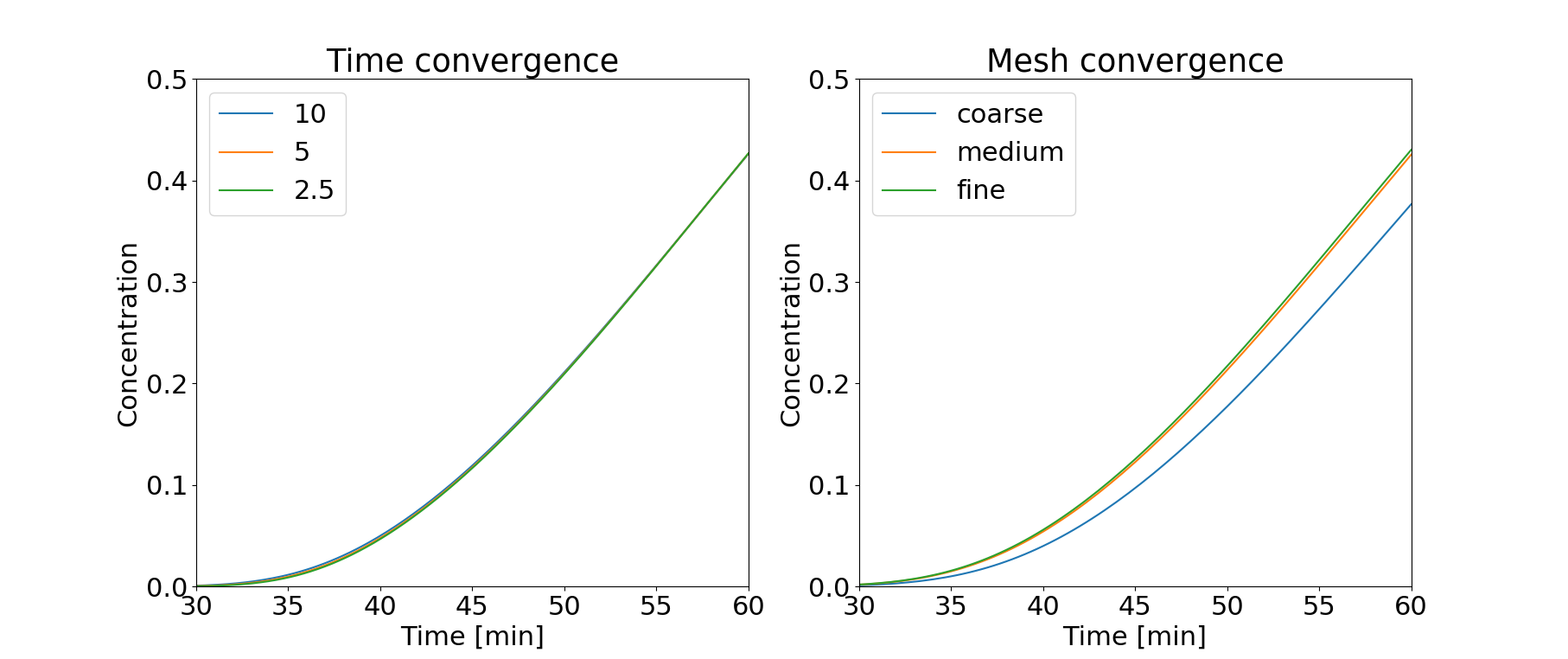

Supplement: Supplementary file 6 — Supplementary Information 6. [file 41598_2021_95306_MOESM6_ESM.png]

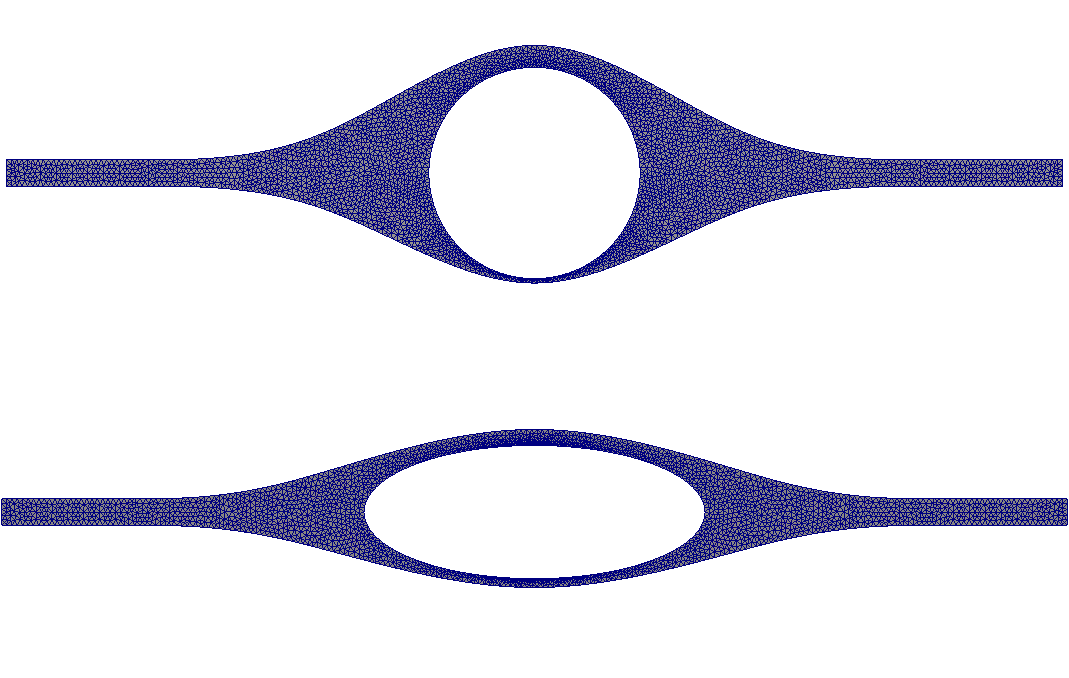

Supplement: Supplementary file 7 — Supplementary Information 7. [file 41598_2021_95306_MOESM7_ESM.png]
