# Supplementary material for: Brain solute transport is more rapid in periarterial than perivenous spaces
Source: Sci Rep. 2021 Aug 9;11:16085. doi: 10.1038/s41598-021-95306-x (PMC8352970; doi:10.1038/s41598-021-95306-x)
Supplement: Supplementary file 8 — Supplementary Legends. [file 41598_2021_95306_MOESM8_ESM.pdf]

Additional file 1 (video): Perivascular flows resulting from a gradual change from periarterial to perivenous space.

Additional file 2 (video): Perivascular flows resulting from a gradual change in PVS widths.

Additional file 3 (video): Concentrations in idealized periarterial and perivenous geometries. A threshold of 0.7 was used to visualize the transport of tracer. Tracers appear earlier around periarterial spaces due to higher convective velocities.

Additional file 4 (video): Concentrations in image-based periarterial and perivenous geometries. A threshold of 0.7 was used to visualize the transport of tracer. Tracers appear earlier around periarterial spaces due to higher convective velocities.

Additional file 5 (figure): Verification of the CFD solver. The left panel shows a colorplot of the simulated velocity in the z-direction. The right panel shows a plot over line from $r = 20$ to $r = 60\mu$m, where r is the distance from the center. Together with our simulation results we show the exact solution $u(r) = \frac{\partial p}{\partial z}\frac{1}{4\mu}\left((r_1^2 - r^2) - (r_1^2 - r_0^2)(\frac{\log(r_1/r)}{\log(r_1/r_0)})\right)$. The peak velocity has a relative error of 0.012.

Additional file 6 (figure): Numerical convergence study. Left: Concentration (AU) in idealized perivenous spaces vs time for different time resolutions $\Delta t = 10, 5, 2.5$ s. Right: Concentration (AU) in idealized perivenous spaces vs time for different mesh resolutions (coarse, medium, fine). The refined meshes were constructed by uniform refinement of the original mesh. Simulation results are reported from the 'medium' mesh and the coarsest time step.

Additional file 7 (figure): The "medium" mesh resolution on geometries A0 and V0 used to perform the simulations
